# Evaluation of short-term outcomes of laparoscopic-assisted surgery for colorectal cancer in elderly patients aged over 75 years old: a multi-institutional study (YSURG1401)

**DOI:** 10.1186/s12893-017-0229-7

**Published:** 2017-03-21

**Authors:** Keisuke Kazama, Toru Aoyama, Tsutomu Hayashi, Takanobu Yamada, Masakatsu Numata, Shinya Amano, Mariko Kamiya, Tsutomu Sato, Takaki Yoshikawa, Manabu Shiozawa, Takashi Oshima, Norio Yukawa, Yasushi Rino, Munetaka Masuda

**Affiliations:** 1 Department of Surgery, Yokohama City University Hospital, 3-9 Fukuura, Kanazawa-ku, Yokohama, 236-0004 Japan; 2 The Department of Gastrointestinal Surgery, Kanagawa Cancer Center, Yokohama, 241-8515 Japan

**Keywords:** Corolectal cancer, Lapaloscopic surgery, Elderly patient, Safety, Feasibility

## Abstract

**Background:**

The short-term outcomes of laparoscopic-assisted surgery for colorectal cancer (LAC) have not been fully evaluated in elderly patients. The aim of this study was to compare the short term surgical outcomes of LAC between the patients older than 75 years and those with non-elderly patients.

**Methods:**

This retrospective multi-institutional study selected patients who underwent LAC between April 2013 and March 2014 at Yokohama City University Hospital and its related general hospitals. The patients were categorized into two groups: elderly patients (>75 years of age: group A) and non-elderly patients (<75 years of age: group B). Surgical outcomes and post operative complications were compared between the two groups.

**Results:**

A total of 237 patients were evaluated in the present study. Eighty-four patients were classified into group A, and 153 into group B. Preoperative clinicopathological outcomes demonstrated no significant differences except for the ASA score. When comparing the surgical outcomes between group A and group B, the rate of conversion to open procedure (3.6% vs 5.2%, *P* = 0.750), median operation time (232 min vs 232 min, *P* = 0.320), median blood loss (20 ml vs 12 ml, *P* = 0.350). The differences were not significantly different in the surgical outcomes. The incidences of > grade 2 post operative surgical complications were similar between two groups ((19.0% vs 15.7%, *p* = 0.587). No mortality was observed in this study. The length of postoperative hospital stay was also similar (10 days vs 10 days, *p* = 0.350).

**Conclusions:**

The present study suggested that LAC is safe and feasible, regardless of the age of the patient, especially for elderly patients who may be candidates for colon cancer surgery.

## Background

Every year, more than 1.36 million people were diagnosed as colorectal cancer (CRC) world-wide, and CRC is the third most frequent cancer-related cause of death [1, 2]. Complete resection is essential for the cure of CRC.

The use of laparoscopic-assisted surgery for CRC (LAC) was first described by Redwine and Schlinkert in 1991 [3, 4]. Since then, the numbers of cases of LAC have been increasing gradually. Some randomized controlled studies demonstrated the acceptable oncologic outcomes and the advantages of this procedure, as compared with open colorectomy (OC), including reduced amounts of operative blood loss and pain, earlier recovery of bowel activity and resumption of oral intake and shorter hospital stays [1, 5–15].

On the other hands, the previous randomized studies excluded the patients over the age of 75 [15, 16] or include only small population of them [1, 5–12]. Therefore, the safety and feasibility of LAC for elderly patients are still unclear. Generally, the elderly patients often have co-morbidities and age-related physiological problems that can lead to greater postoperative complications than non-elderly patients [17]. When considering LAC in elderly patients, the specific effects of its procedure, such as Trendelenburg position, pneumoperitoneum, and long operative time may result in poor outcomes that were unlikely to be seen in no-elderly population [18].

The aim of this study was to compare the short term surgical outcomes of LAC and evaluate the safety and feasibility of LAC and compared them with non-elderly patients using multi institute data.

## Methods

### Patients

This is retrospective multi-institutional study. Patient’s record was retrieved from the collected database of Yokohama City University, Department of surgery and its affiliated institutions between March 2013 and April 2014. The inclusion criteria were as follows; (i) histologically proven colorectal cancer, (ii) the patients over 20 years old, (iii) received laparoscopic surgery with lymph node dissection for colorectal cancer as primary treatment. The patients were categorized into two groups: elderly patients (>75 years of age: group A) and non-elderly patients (<75 years of age: group B).

### Surgical procedure

In principle, LAC was performed by 5 ports method under general and epidural anesthesia. Functional end to end anastomosis (FEEA) was performed for right-sided colectomy, and double stapling technique (DST) was performed for left-sided colectomy and rectum anterior resection. The number and position of intra-abdominal drainage tubes were determined by each surgeon. Pathological staging was carried out according to the UICC classification. The appropriate length of resection and the levels of lymph node dissection were generally determined by the Japanese Society for Cancer of the Colon and Rectum (JSCCR) Guidelines 2010 [19]. Moreover, the epidural anesthesia was routinely performed for all patients.

### Evaluation of operative morbidity and mortality

The grade 2–5 postoperative complications (according to the Clavien-Dindo classification (Table 1) that occurred during hospitalization and/or within 30 days after surgery were retrospectively determined from the patient’s records [20]. Grade 1 complications were not evaluated, to exclude the possibility of a description bias in the patient’s records.

**Table 1:** Clavien-Dindo classification

Grade	Definition
Grade I	Any deviation from the normal postoperative course without the need for pharmacological treatment or surgical, endoscopic, and radiological interventions Allowed therapeutic regimens are: drugs as antiemetics, antipyretics, analgetics, diuretics, electrolytes, and physiotherapy. This grade also includes wound infections opened at the bedside.
Grade II	Requiring pharmacological treatment with drugs other than such allowed for grade I complications
Grade III	Requiring surgical, endoscopic or radiological intervention Intervention not under general anesthesia Intervention under general anesthesia
Grade IIIa	
Grade IIIb	
Grade IV	Life-threatening complication (including CNS complications)* requiring IC/ICU management Single organ dysfunction (including dialysis) Multi organ dysfunction
Grade IVa	
Grade IVb	
Grade V	Death of a patient
Suffix “d”	If the patient suffers from a complication at the time of discharge, the suffix “d” (for disability) is added to the respective grade of complication. This label indicates the need for a follow-up to fully evaluate the complication.

*Brain hemorrhage, ischemic stroke, subarrachnoidal bleeding, but excluding transient ischemic attacks, *CNS* central nervous system, *IC* intermediate care, *ICU* intensive care unit

### Statistical analyses

The values are expressed as the median and range. The statistical analyses were performed using the chi-square test or the Wilcoxon signed-rank test. A *P* value of less than 0.05 was considered to indicate statistical significance. The SPSS software package (v12.0 J Win, SPSS, Chicago, IL) was used for all statistical analyses. R-category and extent of dissection were determined by the Japanese Classification of Colorectal Carcinoma 8^th^ [21].

### Ethics

The study was approved by the each Institutional Review Board of each hospital.

## Results

### Demographic and clinical characteristics

A total of 237 patients underwent LAC between 2013 and 2014 at nine institutions. Eighty-four patients were classified into group A, and 153 into group B. The background of the patients is summarized Table 2. There were no patients were received chemoradiation therapy before surgery in the present study. The American Society of Anesthesiology (ASA) score was significantly worse and the incidence of the colon cancer marginally higher in Group A than in Group B (*p* < 0.001 and *p* = 0.058, respectively). On the other hands, similar values were observed in body mass index and preoperative laboratory data findings.

**Table 2:** Demographic and clinical characteristics

Factor	Group A (Age ≥ 75) (*n* = 84) (%)	Group B (Age < 75) (*n* = 153) (%)	*P* value
Gender, n (%)			1.000
Male	46 (54.8)	83 (54.2)	
Female	38 (45.2)	70 (45.8)	
ASA score, n (%)			<0.001
1	6 (7.1)	46 (30.1)	
2	72 (85.7)	100 (65.4)	
3	6 (7.1)	7 (4.6)	
Charlson score (range)	0 (0–4)	0 (0–8)	0.659
Body mass index (range) (kg/m^2^)	21.9 (12.7–32.1)	22.1 (15.0–37.7)	0.167
Tumor location, n (%)			0.058
Right-sided colon (cecum, ascending, transverse)	36 (42.9)	49 (32.0)	
Left-sided colon (descending, sigmoid, rectosigmoid)	36 (42.9)	63 (41.2)	
Rectum, Proctodeum	12 (14.3)	41 (26.8)	

### Surgical and pathological findings

The operative details and pathological data were shown in Table 3. When comparing the surgical outcomes between group A and group B, the rate of conversion to open procedure (3.6% vs 5.2%, *P* = 0.750, one patient was bleeding and two were technical difficulties for securing a field of view over surgical sites in the elderly patients, while three patients were bleeding, one was technical difficulty, and others were metastasis or suspected direct invasion to other organ site in the non-elderly patients), median operation time (232 min vs 232 min, *P* = 0.320), median blood loss (20 ml vs 12 ml, *P* = 0.350). The differences were not significantly different in the surgical outcomes. Moreover, there were 12 patients having a stoma in non-elderly patients and 4 patients having a stoma in elderly patients. There was no statically difference between two groups. On the other hands, the rate of D3 Lymph node dissection was higher in group B (56.0% vs. 69.3%, *P* = 0.047). Pathologically, there are no statistically significant differences in T classification, N classification and UICC pathological Stage of the two groups.

**Table 3:** The operative details and pathological data

Factor	Group A (*n* = 84) (%)	Group B (*n* = 153) (%)	*p*-value
Duration of surgery, min (range)	232 (99–590)	232 (123–590)	0.318
Blood loss, ml (range)	20 (2–669)	12 (2–1143)	0.353
Lymph node dissection, n (%)			0.047
D1 or D2	37 (44.0)	47 (30.7)	
D3	47 (56.0)	106 (69.3)	
Completeness of resection, m (%)			0.130
R0	79 (94.0)	143 (93.5)	
R1	2 (2.4)	0 (0.0)	
R2	3 (3.6)	10 (6.5)	
Conversion to open surgery, n (%)	3 (3.6)	8 (5.2)	0.751
p T classification, n (%)			0.881
Tis	13 (15.4)	22 (14.5)	
T1	11 (13.1)	30 (19.9)	
T2	14 (16.7)	27 (17.9)	
T3	36 (42.9)	57 (37.7)	
T4	10 (11.9)	15 (10.0)	
p N classification, n (%)			0.218
N0	62 (73.8)	99 (64.7)	
N1	15 (17.9)	43 (28.1)	
N2	7 (8.3)	11 (7.2)	
UICC p Stage, n (%)			0.548
Stage0	6 (7.1)	8 (5.2)	
StageI	30 (35.7)	57 (37.3)	
StageII	25 (29.8)	30 (19.6)	
StageIII	19 (22.7)	44 (28.7)	
StageIV	4 (4.8)	13 (8.5)	

### Postoperative complications

Postoperative outcomes and its details were shown in Table 4. The incidences of > grade 2 post operative surgical complications were similar between two groups (19.0% vs 15.7%, *p* = 0.587). No mortality was observed in this study. The median Length of postoperative hospital stay is almost the same in the two groups (10 days (6–51) vs. 10 days (5–62)).

**Table 4:** Postoperative outcomes and its details

Factor	Group A (*n* = 84) (%)	Group B (*n* = 153) (%)	*p*-value
Complication, n (%)			
Overall	16 (19.0)	24 (15.7)	0.587
≥ Grade II	13 (15.5)	18 (11.8)	0.427
Grade of complication, n (%)			0.249
0	68 (81.0)	129 (84.3)	
I	3 (3.6)	6 (3.9)	
II	8 (9.5)	6 (3.9)	
IIIa	5 (6.0)	6 (3.9)	
IIIb	0 (0.0)	6 (3.9)	
Mortality, n (%)	0 (0.0)	0 (0.0)	1.000
Length of postoperative stay (day) (range)	10 (6–51)	10 (5–62)	0.347
Type of complication, n (%)			
Surgical site infection	3 (2.0)	5 (6.0)	0.136
Organ site infection	1 (0.7)	1 (1.2)	1.000
Anastomotic leakage	9 (5.9)	2 (2.4)	0.336
Ileus	2 (1.3)	2 (2.4)	0.616
Pneumonia	0 (0.0)	1 (1.2)	0.354
Urinary dysfunction	2 (1.3)	0 (0.0)	0.54
Abdominal incisional hernia	2 (1.3)	0 (0.0)	0.54
Bleeding	2 (1.3)	0 (0.0)	0.54
Venous thrombosis	0 (0.0)	1 (1.2)	0.354
Delirium	0 (0.0)	2 (2.4)	0.125
Diarrhea	2 (1.3)	2 (2.4)	0.616
Urinary tract infection	1 (0.7)	0 (0.0)	1.000

In concerning the details of complications, in group A, anastmotic leakage was most likely to be observed (5.9%), and surgical site infection was the major complication observed in group B (6.0%). Urinary dysfunction, urinary tract infection, abdominal incisional hernia, and postoperative bleeding were seen only in the group A, on the other hand, venous thrombosis, pneumonia and delirium in group B.

## Discussion

The present study showed that the short-term outcomes, including overall postoperative complication rates, mortality rates were similar between elderly and non-elderly patients. Furthermore, the details of the perioperative course and length of hospital stay were similar between the groups. Therefore, our results suggest that LAC is a safe and feasible regardless of the age of the patient.

The overall complication rates were 19.0% in Group A and 15.7% in Group B. There were no statistically significant differences (*P* = 0.587). Anastomotic leakage (5.9%) and surgical site infection (2.0%) were main complications in Group A. Surgical site infection was the most frequently diagnosed complication in Group B, followed by ileus, leakage, diarrhea and delirium. Moreover, no mortality was observed in both groups. Similar results were observed in previous reports. For example, Inoue et al. evaluated efficacy and safety of laparoscopic surgery in elderly patients with colorectal cancer [22]. They compared the differences between elderly patients (aged >75 years, *n* = 48) and non-elderly patients (aged <75 years, *n* = 100) and evaluated the demographics and disease-related operative and prognostic data. They found that the postoperative complications were observed 14.0% in less than 75 years of age group and 20.8% in 75 years or older group (*p* = 0.299). Moreover, the mortality rate was similar between less than 75 years of age group and 75 years or older group (0.7% vs 2.1%, *P* = 0.1475). Chautard et al also reported similar results [23] They conducted a prospective case-matched study to compare outcomes of laparoscopic colorectal surgery in elderly (>or = 70 years) and younger (<70 years) patients. They found that the postoperative complications were observed 26% in less than 70 years of age group and 32% in 70 years or older group (*p* = 0.299). There was no mortality. On the other hands, Kang et al. evaluated the short-term outcomes of LAC in 578 patients and divided them into 2 groups. When using cutoff value of 75 years old, overall comorbidity rate is similar between elderly and non-elderly patients (20.0% vs. 26.5%, *p* = 0.149). However, the severity of the complications were tend to be higher in elderly group (Clavien-Dindo classification of I/II/III/IV were 6.2%/9.6%/3.1%/1.0% in non-elderly group, 4.1%/18.4%/3.1%/1.0% in elderly group) [24]. These data might suggest that when complications occur in elderly patients, they rapidly increase in severity. The further study should be focus on this issue.

In the present study, the perioperative course, such as operation time, intraoperative blood loss, and length of the hospital stay, was similar between two groups. Previous reports demonstrated similar results [22–33]. On the other hands, the details of surgical procedure were different between the elderly and non-elderly patients. First, the incidence of the D3 lymph node dissection was lower in the elderly patients than in the non-elderly patients. D3 LN resection is essential to achieve better oncologic prognosis. Actually, almost all advanced tumor stage patients in the elderly patient group received D3 LN resection. On the other hands, although 50% of the non-elderly patient was diagnosed advanced tumor stage, 70% of non-elderly patients were received D3 LN resection in the present study. Therefore, the surgeons might avoid performing D3 lymph node dissection in elderly patients. Similar trends were observed in the previous reports. For example, Mukai et al reported short-term outcomes of octogenarian patients who underwent LAC [26]. In their study, postoperative comorbidity rare was acceptable (13.6%), but the D3 rate was as much as 48%. Second, the location of the tumor was different. The incidence of the rectum was 14.3% in the elderly patients and 26.8% in the non-elderly patients. Surgeons also avoid performing laparoscopic rectum surgery in elderly patients, because the postoperative complication of laparoscopic rectum surgery is higher than those of laparoscopic colon surgery. Actually, in the past randomized controlled study, postoperative complication rates of LAC were reported 14–34% in colon cancer, while 40–44% in rectal cancer [1, 5–13]. Moreover, although there were not significant differences, anastomotic leak and conversion rate were trend to high in non-elderly patients than elderly patients. Difference of tumor location between two groups might be affected for the results.

When interpreting our results, special attention is required, because there are several potential limitations associated with this study. First, this study was a retrospective study with a limited sample size, despite patients data were collected in multi-institutions. For example, enhanced recovery after surgery (ERAS) protocol may improve length of stay and morbidity of the patients. However, we did not collect the ERAS protocol data. Further study will focus on this issue. Second, the definition and classification of morbidity were different from those used in the previous studies. These differences might have affected the results and discussion. Third, we could not deny the possibility that the elderly people in the present study might be selected and fit for surgery. In the present study, the surgical indication was determined by physicians, including an anesthesiologist, who took into consideration the activities of daily living, performance status, medical history, physical examinations, and organ functions, as is done in general community hospitals. However, there is a possibility that only patients with good status were selected because the morbidity after colorectomy has been reported to range from 10 to 20%, and the complications are sometimes fatal. Actually, although the ASA physical status was significantly different between two groups, Charlson score and body mass index were similar between the two groups. Moreover, the surgical operations are very heterogeneous and different between the two groups. The number of rectal resections is significantly higher in non-elderly patients and this is a bias for duration of surgery, minor and major complications. Considering these limitations of the present study, further studies should focus on which patients are candidates for LAC and will do well after LAC.

In summary, the short-term outcomes of LAC were almost equal in the elderly and non-elderly patients in this study. Therefore, it is unnecessary to avoid LAC in elderly patients who may be good candidates for LAC, simply because of their age.

## Conclusion

The short-term outcomes of safety and feasibility of LAC were almost equal in the elderly and non-elderly patients. Therefore, it is unnecessary to avoid LAC in elderly patients who may be good candidates for LAC, simply because of their age.
